# A method for spatial interpretation of weakly supervised deep learning models in computational pathology

**DOI:** 10.1038/s41598-025-04043-y

**Published:** 2025-06-05

**Authors:** Abhinav Sharma, Bojing Liu, Mattias Rantalainen

**Affiliations:** Department of Medical Epidemiology and Biostatistics, Karolinska Institutet, Stockholm, 17177 Sweden

**Keywords:** Deep learning, Image analysis, Computation pathology, Weakly supervised learning, Spatial interpretability, Machine learning, Statistical methods

## Abstract

Deep learning enables the modelling of high-resolution histopathology whole-slide images (WSI). Weakly supervised learning of tile-level data is typically applied for tasks where labels only exist on the patient or WSI level (e.g. patient outcomes or histological grading). In the weakly supervised learning context, there is a need for a methodology that facilitates the identification of the precise spatial regions in WSI that drive the prediction of the slide label. Such information is also needed for any further spatial interpretation of predictions from such models. We propose a novel method, Wsi rEgion sElection aPproach (WEEP), for model interpretation. It provides a principled yet straightforward way to establish the spatial area of WSI required for assigning a particular prediction label. We demonstrate WEEP on a binary classification task in the area of breast cancer computational pathology. WEEP facilitates the identification of spatial regions in WSI that are driving the decision making of a particular weakly supervised learning model, which can be further visualised and analysed to provide spatial interpretability of the model. The method is easy to implement, is directly connected to the model-based decision process, and offers information relevant to both research and diagnostic applications.

**Supplementary Information:**

The online version contains supplementary material available at 10.1038/s41598-025-04043-y.

## Background

Deep learning-based models have demonstrated high performance in a range of prediction tasks in the digital pathology domain. Due to hardware constraints, a common strategy is to divide the gigapixel-size whole slide images (WSIs) into smaller patches (i.e. tiles), which are subsequently modelled by e.g. deep Convolutional Neural Network (CNN) models^1-3^. In situations where labels are only available on the WSI level, either due to the absence of pixel-level annotations or the presence of labels exclusively on the WSI level (e.g. patient outcomes, time-to-event, treatment response), weakly supervised learning is often applied in the domain of computational pathology^4^. This type of precision pathology applications^5^ are of high value in medicine, as artificial intelligence has the potential to go beyond what a human expert can see in a histopathology specimen and thus extract and provide new or additional information with high clinical relevance.

Weakly supervised learning has demonstrated broad success in precision pathology and computational pathology in general, also in the presence of label noise. However, the interpretation of these models is not straightforward, despite the importance of the interpretation in both research applications and clinical decision support applications. Conventional tools for the interpretation of deep CNN models, such as class activation maps (CAM)^6^ and Gradient-weighted class activation maps (Grad-CAM)^7^, are focused on pixel-level local interpretability (within tile), which do not have a direct interpretation with respect to the predicted label at the WSI level (across all tiles). Recently, trainable attention-based neural networks^8^ have been introduced in histopathology image analysis^9^. However, the attention weights provide the ranking of tiles but but it is hard to determine an objective rule without the link to actual predictions to establish the threshold directly to the attention weights which can determine the set of tiles (i.e. area) required for a particular classification label.

In precision pathology, spatial interpretability has the potential to highlight areas (at tile level) with the most relevant tissue morphology for the particular modelling task at hand. Especially in the context of weakly supervised deep learning models based on tile-level data, methodology for spatial interpretability, defined by the association of spatial regions in WSI linked to the slide-level prediction problem, has not been previously described.

To address this problem, we propose a novel methodology, the Wsi rEgion sElection aPproach (WEEP), enabling ascertainment of WSI regions that are required for a positive classification label at the slide-level, in the common scenario of tile-based weakly supervised learning. The WEEP output has a direct connection to the prediction problem, and results are conditional upon the specific model applied and its parameters and reveal the specific tiles and the spatial area that drive the assignment of the classification in each WSI. The approach is easy to implement and has a direct interpretation with respect to the prediction problem and is therefore relevant in both research and clinical application contexts.

To illustrate the method, we apply WEEP to a binary classification task, the classification of histological grade 1 and 3 in breast cancer. Histological grade is a well-established histomorphology-based prognostic marker used to determine the prognosis and treatment planning for the breast cancer patients^10^. Deep learning based binary classification of histological grade using MIL-based pooling for tile-to-slide level aggregation has been demonstrated in several studies previously^11-14^. The selected regions from WEEP can also contribute to understanding the WSI areas and morphological patterns for novel histo-morphological biomarker discovery. In the clinical setting, visualisation of the selected regions from WEEP can enable the clinical pathologist to confirm and assess the regions when signing out a case where AI-based decision support has been used, for example, to identify risk stratification of patients. Similarly, the method can be further extended to other precision pathology tasks that include survival prediction, treatment prediction among others.

In this study, we describe the WEEP methodology and apply it to identify classification-essential WSI regions from three different tile-to-slide level aggregation functions: a trivial mapping function based on the 75th percentile of tile-level prediction scores from CNN models, a tile-level attention scores from a trainable attention-based model, and a transformer-based aggregation model.

## Methods

### WEEP method description

Multiple Instance learning (MIL) is one of the standard frameworks for weakly supervised learning problems in computational pathology^15^. In a standard MIL framework, a bag (represented by the WSI) contains instances (represented by the tiles), and if at least one of the instances is classified as positive then the whole bag is classified as positive. However, in real-world computational pathology applications, the bag level label is typically assigned based on a function that accounts for more than a single tile in order to achieve optimal prediction performance. The MIL framework can be divided into two steps: first, the tile-level feature representations/prediction scores are obtained from a base CNN model and second: these feature representations/prediction scores are aggregated to provide the WSI prediction score using a tile-to-slide aggregator function or model. Different tile-to-slide level aggregator functions have been used in different weakly supervised classification scenarios^16^.

WEEP exploits a fundamental property of MIL models applied to tile-level instances of the histopathology WSIs. Specifically, we assume the existence of a model that provides tile-level predictions, that allow the ranking of tiles with respect to the prediction task, based on e.g. predicted class probabilities, such that two instances x_i_ and x_j_, p(class = 1 | x_i_) > p(class = 1| x_j_) implies x_i_ is more likely than x_j_ to belong to class 1, conditional upon the model and the tile. In the case of a simple tile-to-slide mapping function, e.g. mean, median or 75-percentile, of the tile distribution, we can simply use the tile class probability prediction. However, in cases where we have a second trainable model for slide-level predictions that has attention weights, these weights can also be considered as the ranking metric.

**Fig. 1:**
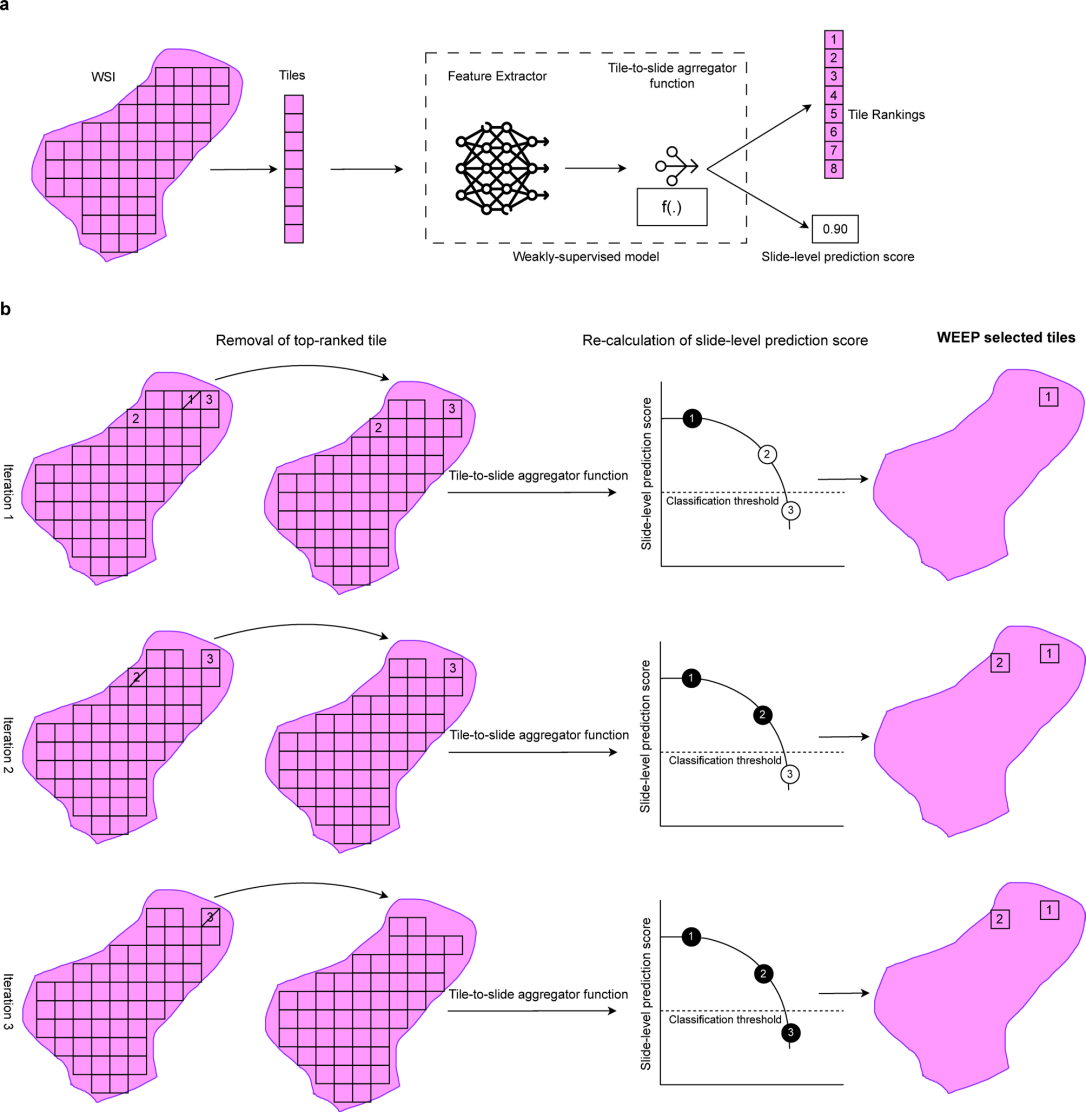
Overview of the WEEP methodology. **a** Demonstrating a weakly-supervised learning scenario for histopathology image analysis. Firstly, whole-slide image (WSI) is divided into small patches called tiles. Then, tiles are provided as input to the weakly-supervised model that is majorly composed of two modules i.e. Feature Extractor and Tile-to-slide aggregator function. The feature extractor module is used to extract low-dimensional representation features from each tile and then tile-to-slide aggregator function (either based on summary statistics or a separate trainable model) is used to provide slide-level prediction score. The tile-to-slide aggregator function utilises the ranking of the tiles based on tile-level prediction scores or optimised attention scores from trainable attention module. **b** Demonstration of the WEEP methodology that applies stepwise backward selection of the tiles based on the ranking retrieved from the tile-to-slide aggregator function. In the first iteration, highest ranked tile is removed from the WSI and then tile-to-slide aggregator function is applied on the remaining tiles to retrieve the slide-level prediction score. The following iterations are followed until the slide-level prediction score reaches below the classification threshold (iteration 3 in the example). The removed tiles are the selected tiles through WEEP that are associated with the positive classification of the WSI.

Irrespective of the ranking metric, a backward selection approach can be applied, which provides guarantee that the selected subset is the maximum set of tiles needed for positive classification on the WSI level. Backward selection is a common strategy that has been used for variable selection in predictive modelling tasks^17^. Here, we apply it to identify the set of instances (tiles) required for a positive classification label (Table 1). In our empirical evaluation results, we consider tile-level predicted class probability as well as attention weights as ranking metrics. The WEEP algorithm allows us to determine the set of tiles directly required to assign the WSI label (Fig. 1).

**Table 1:**
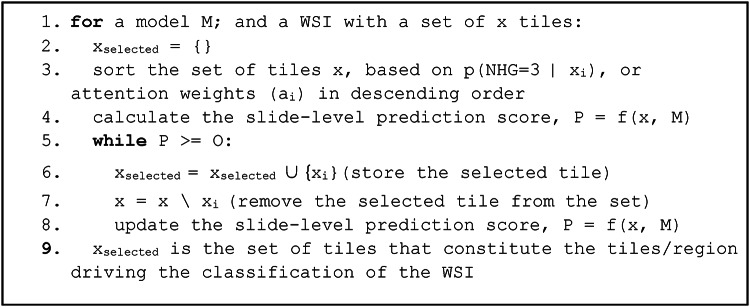
Outline of the steps in the WEEP algorithm. (*O represents the established decision threshold)*

### Study materials

In this study, we included patients from the SöS-BC-4 cohort, collected from the Södersjukhuset (South General Hospital) in Stockholm, Sweden from the year 2012–2018. One Hematoxylin and Eosin (H&E) stained WSI scanned at 40X magnification level was considered from each patient. WSI preprocessing steps were followed as described in^13^. We only included tiles predicted as invasive cancer (tile size: 598 × 598 pixels at 20x resolution) in further analyses. The study has approval from the Swedish Ethical Review Authority (2017/2106-31, with amendments 2018/1462-32 and 2019–02336). The study was performed in accordance with the Declaration of Helsinki. No additional informed consent was required in accordance with ethical approval in this non-interventional collection and analysis of data from patient records.

The models were optimised and validated on the subset of SöS-BC-4 (*N* = 1695) using 5-fold cross-validation (cv). The dataset was split into CV training and CV test set for each cv fold. Further, the CV training set was split into the training set, and the tuning set^14^. Each data split was stratified by the clinical NHG. The SöS-BC-4 data split is shown in Supplementary Fig. 1.

### Description of the four weakly-supervised modelling strategies

In this study, we included four weakly supervised modelling strategies. In the first modelling strategy, we considered the Resnet-18 CNN model architecture^18^ as the tile-level classification model. The tiles were assigned weak slide-level label and pretrained model from Imagenet was used to initialise the model weights^19^ and was fine-tuned as a supervised binary histological grade 1 vs. 3 classification model on the feature extractor training set (Supplementary Fig. 1). The 75th percentile of the tile-level prediction scores from the CNN models was used to provide the slide-level prediction score as the first tile-to-slide aggregation function^13^.

For the second modelling strategy, the tile-level feature vectors were extracted from the average pooling layer of the fine-tuned Resnet-18 models from the first modelling strategy, in the attention module training set and tuning set (Supplementary Fig. 1). The trainable attention-based MIL (atten-MIL) model was considered as the second tile-to-slide aggregation function. It includes the attention layer that is a trainable layer inspired by^15^, which optimises an attention weight to each tile-level feature vector and provides a slide-level prediction score by performing a weighted average of all tile-feature vectors belonging to that slide. The third modelling strategy included extraction of features for the CV training set using the publicly available foundation model called UNI^20^. The transformer-based MIL model called TransMIL^21^ was considered as the tile-to-slide aggregator function. The fourth modelling strategy included atten-MIL as the tile-to-slide aggregation function using the features extracted from UNI. Optimisation of the four modelling strategies is described in the Supplementary.

### Validation on the CV test set

The optimised weakly-supervised models were validated on the CV test set in each CV fold. We further aggregated the slide-level prediction scores from the five CV test sets and evaluated the optimal classification threshold to classify the WSIs as Grade 3 and 1 using the Youden’s statistics.

### Quantitative and visual analysis of selected regions in different modelling strategies

We considered the assessment of the WSI regions contributing to the classification of the histological grade 3 WSIs (*n* = 543). We demonstrated the iterative backward selection approach of the WEEP methodology as the line plot (referred to as the *WEEP plot*). Further, we observed the distribution of the percentage of selected tiles for each WSI in the CV test set using the histogram plots. The selected tiles using WEEP for the example WSIs were visualised over the tumour mask on the low-resolution WSI as the binary mask. All the plots were created using the package matplotlib (v.3.6.2)^22^ in python (v.3.10.8).

## Results

### Evaluating the selected regions from different modelling strategies

**Fig. 2:**
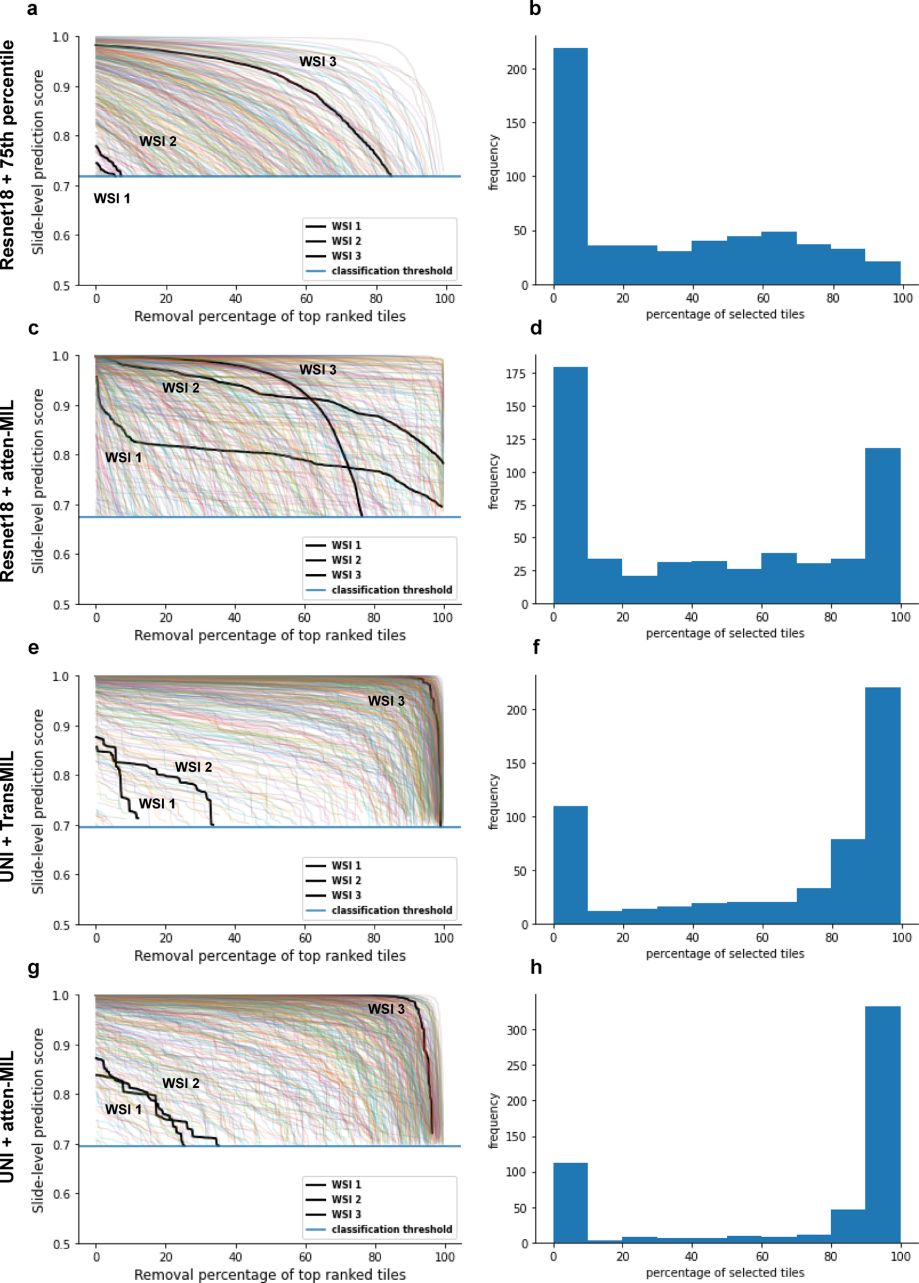
Application of WEEP to a binary classification problem. We have randomly selected and highlighted three clinical NHG 3 WSIs with each WSI randomly selected from the ranges < 20%, > 20% & <80% and > 80% of the selected tiles through WEEP using the UNI as feature extractor and TransMIL as the tile-to-slide aggregation model. They are highlighted (black) in plots **a**, **c**,** e**,** g**. **a**,** c**,**e**,** g** WEEP plot showing the change in slide-level prediction score with the step-wise backward removal of top-ranked tiles until the slide-level prediction score reaches the classification threshold. **a**, ranking of the tiles was based on tile-level prediction scores from the fine-tuned Resnet-18 model and slide level score was determined using the 75th percentile tile-to-slide aggregation function. **c**, ranking of the tiles was based on the attention scores from the attention-based MIL (atten-MIL) model with fine-tuned Resnet-18 as the feature extractor. **e**, ranking of the tiles was based on the attention scores from the TransMIL model with UNI as the feature extractor and **g**, ranking was based on attention scores from atten-MIL model with UNI as the feature extractor. **b**,** d**,**f**,** h** Distribution of the percentage of selected tiles for each WSI from WEEP when applied to the: **b** tile-level prediction scores with 75th percentile tile-to-slide aggregation function, **d** attention scores from the attention-based MIL (atten-MIL) model with fine-tuned Resnet-18 as the feature extractor, **f** attention scores from the TransMIL model with UNI as the feature extractor, **h** attention scores from the atten-MIL model with UNI as the feature extractor.

First, we applied WEEP to analyse two different tile-to-slide aggregation functions for the binary classification problem of NHG 1 vs. 3, and visualised the results as the WEEP plot (Fig. 2a, c, e and g). The results reveal the trajectories of the WEEP backward selection and the corresponding WSI-level prediction score, the horizontal line shows the decision boundary established on the full dataset. Next, we investigated the distribution of the proportion of tiles that were selected by WEEP in each WSI (Fig. 2b, d, f and h). For the ResNet-18 model with 75th percentile tile-to-slide aggregator function, a mean percentage of 32.34% (95% CI: 29.69 − 34.99%) was observed as the average percentage of tiles contributing to the histological grade 3 classification in each slide. For the ResNet-18 model with the attention module, we observed the mean percentage of selected tiles as 44.97% (95% CI: 41.69 − 48.26%). For the UNI as the feature extractor, we observed the mean percentage of selected tiles as 63.88 (95% CI: 60.70 − 67.07%) and 71.97% (95% CI: 68.66 − 75.29%), with the atten-MIL and the TransMIL model respectively. Further, we have reported the classification performance of the four weakly-supervised modelling examples in the Supplementary Fig. 2 and the change in the distribution of percentage of selected tiles for varying thresholds at different model sensitivities are reported in Supplementary Fig. 3.

## Visualisation of the selected regions from different modelling strategies

**Fig. 3:**
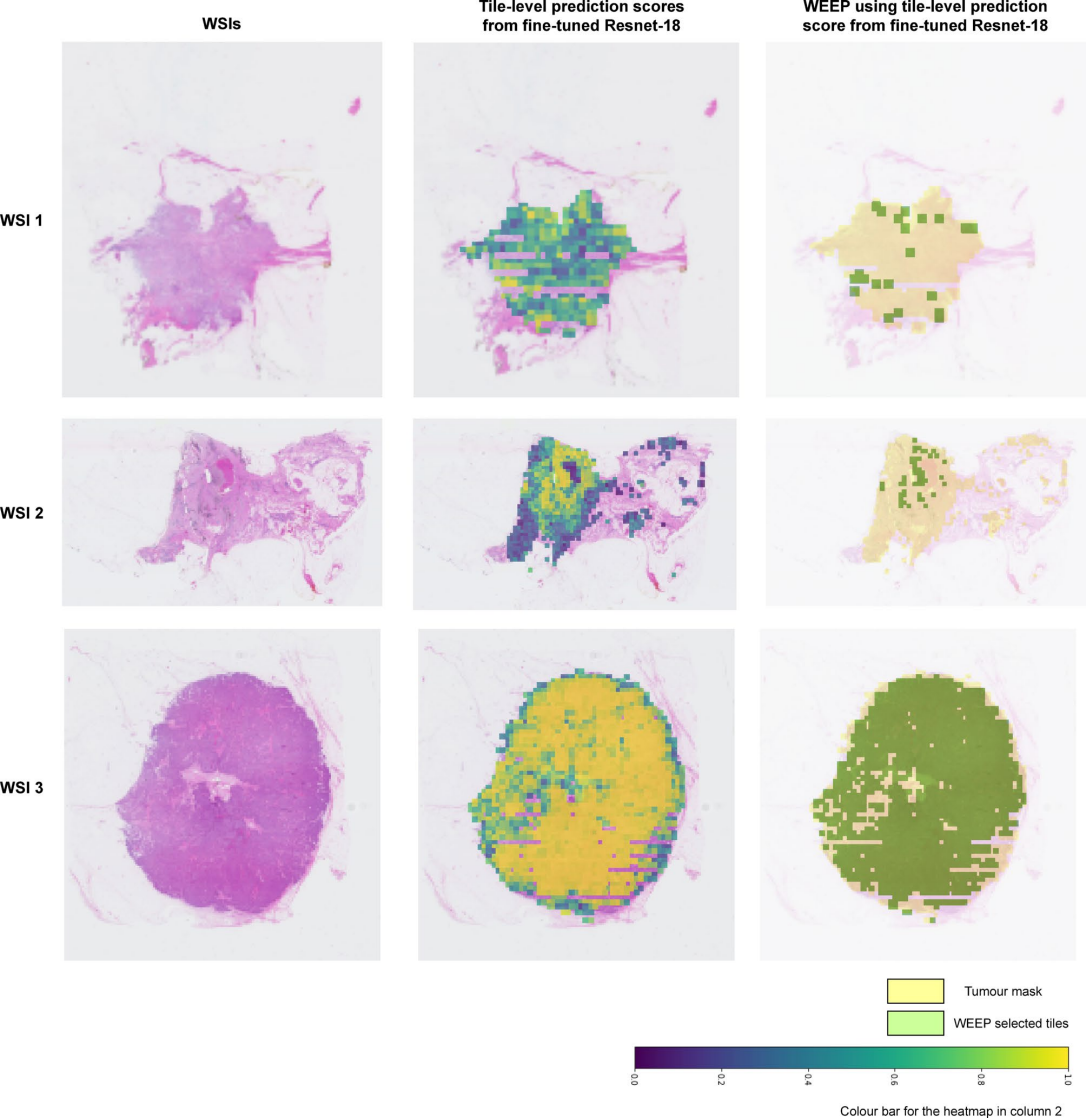
Binary masks of the selected tiles through WEEP over the tumour mask on the original WSI. Randomly selected three clinical NHG 3 WSIs from Fig. 1 have been demonstrated here. The second column includes the heatmap of tile-level scores overlayed on the WSI. The third column represents the binary masks of the selected tiles by applying WEEP to the tiles ranked by tile-level prediction scores using the fine-tuned Resnet-18 model.

To provide an example of the resulting spatial interpretation, we visualised the WEEP selected regions using tile-level prediction scores from fine-tuned Resnet-18 model with 75th percentile as tile-to-slide aggregation function as the modelling strategy. (Fig. 3). Revealing the spatial localization and WSI regions that are directly linked with the classification of histological grade 3 WSIs. The visualisation of WEEP selected regions using attention scores from TransMIL and atten-MIL as tile-to-slide aggregation models with UNI and fine-tuned Resnet-18 as tile-level feature extractors are available in Supplementary Fig. 4.

## Discussion

In this study, we proposed WEEP, a method that provides a spatial tile-level interpretation of deep learning models for prediction modelling of histopathology whole slide images. WEEP is directly linked to the WSI level prediction by a deep learning model and is based on the ranking of the tile-level predictions together with the application of a backward selection strategy to define the subset of tiles driving the assignment of a classification label.

WEEP was demonstrated on CNN and ViT-based models to classify histological grade 1 vs. 3 in invasive breast cancer patients and evaluated on three different tile-to-slide aggregation functions i.e. 75th percentile of the tile level prediction scores, the trainable attention-based MIL (atten-MIL) model and transformer-based MIL model (TransMIL). We demonstrated the methodology and visually explored the selected regions in the histological grade 3 patients.

CAM-based approaches are common for pixel-level image interpretation but lack a direct connection to the models’ assignment of predictions and decision-making. While the pixel-level information can be of importance to subjectively interpret the model and image patterns learned, areas that are directly linked to how classification labels are assigned are not provided by CAM-based methodologies. Moreover, different CAM-based approaches provide different saliency maps for the same CNN model, which raises questions about the reliability of these methods in offering robust interpretation, especially when semantic pixel-level annotations are not available by definition (e.g. as is the case for prognostic models or other patient outcome-oriented tasks) so that the methods cannot be validated by ground truth annotations^23^. In our application domain of histopathology images modelled as tiles, there is also a direct limitation as CAM-based methods only offer interpretability on individual tiles, making it even harder to identify areas (tiles) across multiple instances in a single WSI that are relevant for assignment of the class label^24^.

MIL-based methods utilising the tile-level class probabilities to aggregate the WSI-level prediction score are hard to interpret since it is unclear to establish the thresholding on tile-level class probabilities to identify the discriminatory tiles in the WSI. Occlusion Sensitivity Analysis (OSA) have been reported to find discriminatory regions locally (in the tiles) by applying the moving mask in the different regions of the tiles and observing the model’s sensitivity (tile-level prediction score) to the different masked regions in a tile^25^. It provides an approach for the local interpretability of the selected tiles if needed but does not have a direct association with the WSI-level classification label. Secondary trainable models for tile-to-slide aggregation like the attention-based approach by Lu et al. provide attention weights to directly visualise the highly discriminatory tiles involved in the classification of the WSI^15^. However, precise identification of the regions that are needed for the slide-level classification is not possible.

In the specific classification scenario of breast cancer histological grading assessment that includes high inter-observer^26^ and inter-lab variability^27^, pathologists assign the NHG label on the WSI and it is not possible to obtain well-defined pixel/tile level annotation by definition. To address this problem, multiple MIL-based weakly-supervised deep learning-based approaches have been developed especially for the histological grade 1 vs. 3 binary classification from the H&E WSIs^11-14^. However, it is important to understand the underlying regions recognised by the models and here, the WEEP methodology can be potentially beneficial in providing regions of interest that are directly associated with the classification of the WSI. It is important to note that the WEEP identifies the minimal areas of the WSI that are needed for a particular classification conditional upon the model. If the WEEP selected area is excluded, the model would provide a different classification result. It provides a way to identify and visualise the spatial regions in the WSI that drive a particular classification decision conditional upon a specific deep learning model.

WEEP has strengths and limitations. One of the main limitations is that the interpretation is provided on the tile level, whereas CAM-based methods are focused on pixel-level interpretations. Secondly, WEEP utilises the underlying model-based ranking of the tiles, which is a potential constraint as the results are conditional upon the model. The major strength lies in the simplicity and empirical approach to defining the direct association of the tiles to the predicted WSI label. The method is also agnostic to the feature extractor and the tile-to-slide aggregation function if the ranking of the tiles associated with the class label can be retrieved. The method can also be extended to the multiclass and regression problems. In the multiclass scenario, WEEP can be applied to the tile-level class probabilities of the predicted class in the WSI. In the regression objectives, it is possible to apply WEEP until the WSI prediction intersects with the e.g. mean (or median) of the predicted response variable.

## Conclusions

The proposed WEEP method provides a direct selection of tiles and regions that could be utilised to visually interpret the decision-making of such tile-based deep learning classification models. The selected regions can be studied further to understand different tissue morphologies used by the model for classification and could be used to determine novel morphological patterns or confirm the learning of the existing associated morphologies by the deep learning-based model.

## Electronic supplementary material

Below is the link to the electronic supplementary material.

Supplementary Material 1

## Data Availability

Data in the study cannot be made publicly available due to legal constraints. Reasonable access requests to the corresponding author will be considered. The implementation of the WEEP methodology is available at: https://github.com/rantalainenGroup/WEEP.

## Abbreviations

WSI: Whole Slide Image
CNN: Convolutional Neural Network
CAM: Class Activation Maps
Grad-CAM: Gradient-weighted class activation maps
WEEP: Wsi rEgion sElection aPproach
MIL: Multiple Instance Learning
H&E: Hematoxylin and Eosin
CV: Cross-Validation
OSA: Occlusion Sensitivity Analysis
