# Supplementary material for: A method for spatial interpretation of weakly supervised deep learning models in computational pathology
Source: Sci Rep. 2025 Jun 5;15:19804. doi: 10.1038/s41598-025-04043-y (PMC12141739; doi:10.1038/s41598-025-04043-y)
Supplement: Supplementary file 1 — Supplementary Material 1 [file 41598_2025_4043_MOESM1_ESM.pdf]

A method for spatial interpretation of weakly supervised deep learning models in computational pathology

Abhinav Sharma[1], Bojing Liu[1], Mattias Rantalainen[1*]
[1] Department of Medical Epidemiology and Biostatistics, Karolinska Institutet, 17165, Sweden
* corresponding author: mattias.rantalainen@ki.se

Table of content:

Figure 1: The SöS-BC-4 cohort splitting criteria for 5-fold CV

Figure 2: Binary classification performance of the four weakly-supervised modelling strategies

Figure 3: WEEP plots and histograms for different modelling strategies at different classification thresholds corresponding to different model sensitivities: 0.50, 0.80, 0.90, and sensitivity at youden's index, obtained from the ROC curve respectively.

Figure 4: Visualisation of WEEP selected tiles using attention scores from TransMIL and atten-MIL tile-to-slide aggregation models with UNI and fine-tuned Resnet-18 as the tile-level feature extractors.

Figure 5: Application of WEEP on histological grade 1 WSIs.

Optimisation of the weakly-supervised models

The splitting criteria used to optimise the four weakly-supervised modelling strategies is shown in Figure 1. The first two modelling strategies included the fine-tuned Imagenet-pretrained Resnet-18 model as the tile-level prediction model and tile-level feature extractor for secondary Atten-MIL model. Fine tuning of the Resnet-18 was performed using the Feature extractor training set and the tuning set (shown in Figure 1a). The first modelling strategy considered the summary statistics (75th percentile) based aggregation of the tile-level prediction scores from the fine-tuned Resnet-18 models on the CV test test. In the second modelling strategy, Atten-MIL models were optimised on the features extracted from the fine-tuned Resnet-18 models using the attention module training set and tuning set (shown in Figure 1a). In the third and fourth modelling strategies, we considered a publicly available foundation model UNI (1). Atten-MIL and TransMIL models were optmised on the extracted UNI features using the CV training set and the tuning set (shown in Figure 1b).

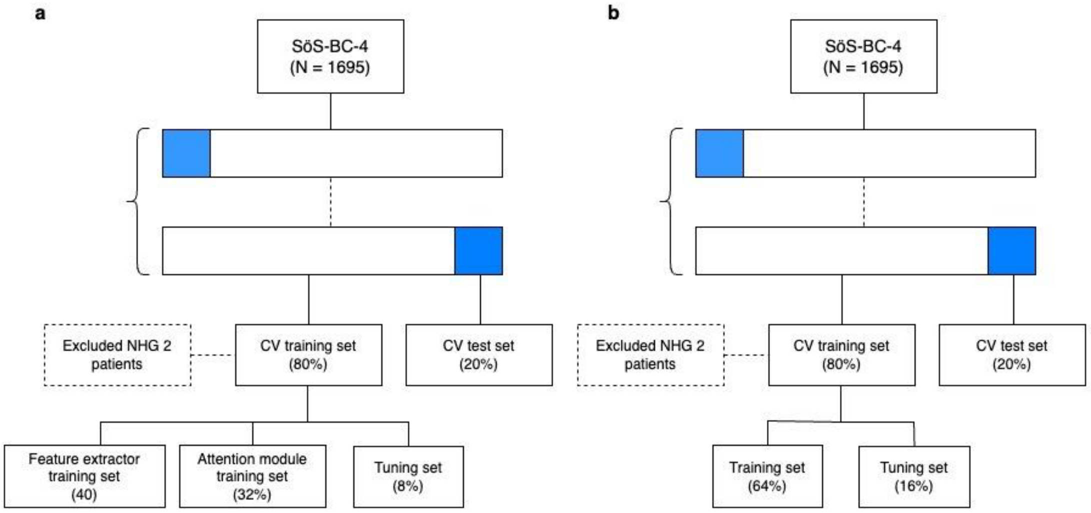

Figure 1: The SöS-BC-4 cohort splitting criteria for 5-fold CV. **a** The splitting criteria for first and second modelling strategies. The Feature extractor training set was used to fine tune the Imagenet-pretrained Resnet-18 model. The fine-tuned Resnet-18 model was used as the feature extractor to optimise the attention-based multiple instance learning (Atten-MIL) model on Attention module training set and the tuning set. **b** The splitting criteria for third and fourth modelling strategies. Publicly available foundation model UNI was used as the feature extractor. The training and tuning set were used to optimise Atten-MIL and transformer-based TransMIL model. Further, detailed cohort description with consort diagram is mentioned in our previous study (2).

In all the modelling strategies, the tuning set was used to monitor the model learning curves, and selection of the best model according to the early stopping criteria. The early stopping criteria was defined as no improvement in tuning loss for the consecutive epochs (patience). For the detailed description of the model optimisation of the fine-tuned Resnet-18 models and the atten-MIL model with fine-tuned Resnet-18 models as the feature extractor (the first two modelling strategies), please refer to our previous publication (2). For the optimisation of

TransMIL and atten-MIL with UNI as the feature extractor, the total number of epochs was set to 100 and the patience was set to 20 with batch size of 1 (third and fourth modelling strategy). The Stochastic Gradient Descent (SGD) (3) was used as the optimizer with learning rate of 1e-5. The models were optimised as the binary classification models to classify NHG 1 vs 3 with cross-entropy loss as the loss function.

Classification performance of the weakly-supervised models

Next, we evaluated the classification performance of the four weakly-supervised modelling strategies that have been used as an example to demonstrate the WEEP methodology. Initially, we observed the AUC values of the binary classifiers in the 5-fold cv test sets of the SöS-BC-4 cohort (Figure 2a) and further, we evaluated the 5-fold cv optimised models on an independent external validation set (SCANB-Lund) (4) (Figure 2b). Note, relatively poor classification performance of the fine-tuned Resnet-18 models (tile-level classifiers and feature extractor) is mainly attributed to the training on smaller dataset (Feature extractor training set in Figure 1a). In the independent external validation set, for each WSI prediction score, we considered the median of the slide-level scores from the 5 cv models to evaluate the classification performance.

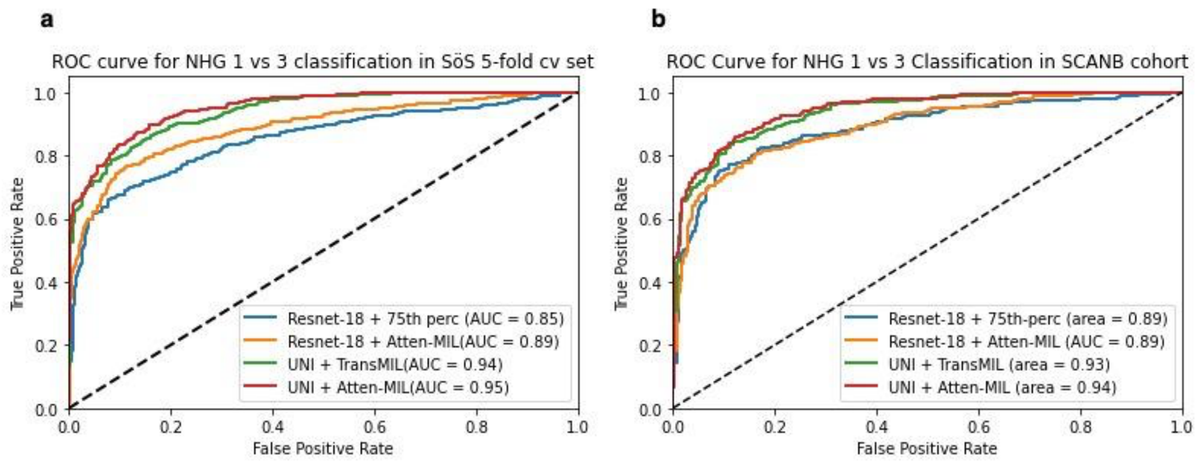

Figure 2: Binary classification performance of the four weakly-supervised modelling strategies. **a** Receiver Operating Characteristic (ROC) curves with the Area Under the Curve (AUC) values on the 5-fold cv test sets. **b** ROC curves with the AUC values on the independent external validation set (SCANB-Lund).

Observation of WEEP plots and selected tile distribution at different classification thresholds

Further, we observed the change in the distribution of percentage of selected tiles using WEEP for different classification thresholds which corresponds to different model sensitivities in the binary histological grade 1vs3 classification (Figure 3).

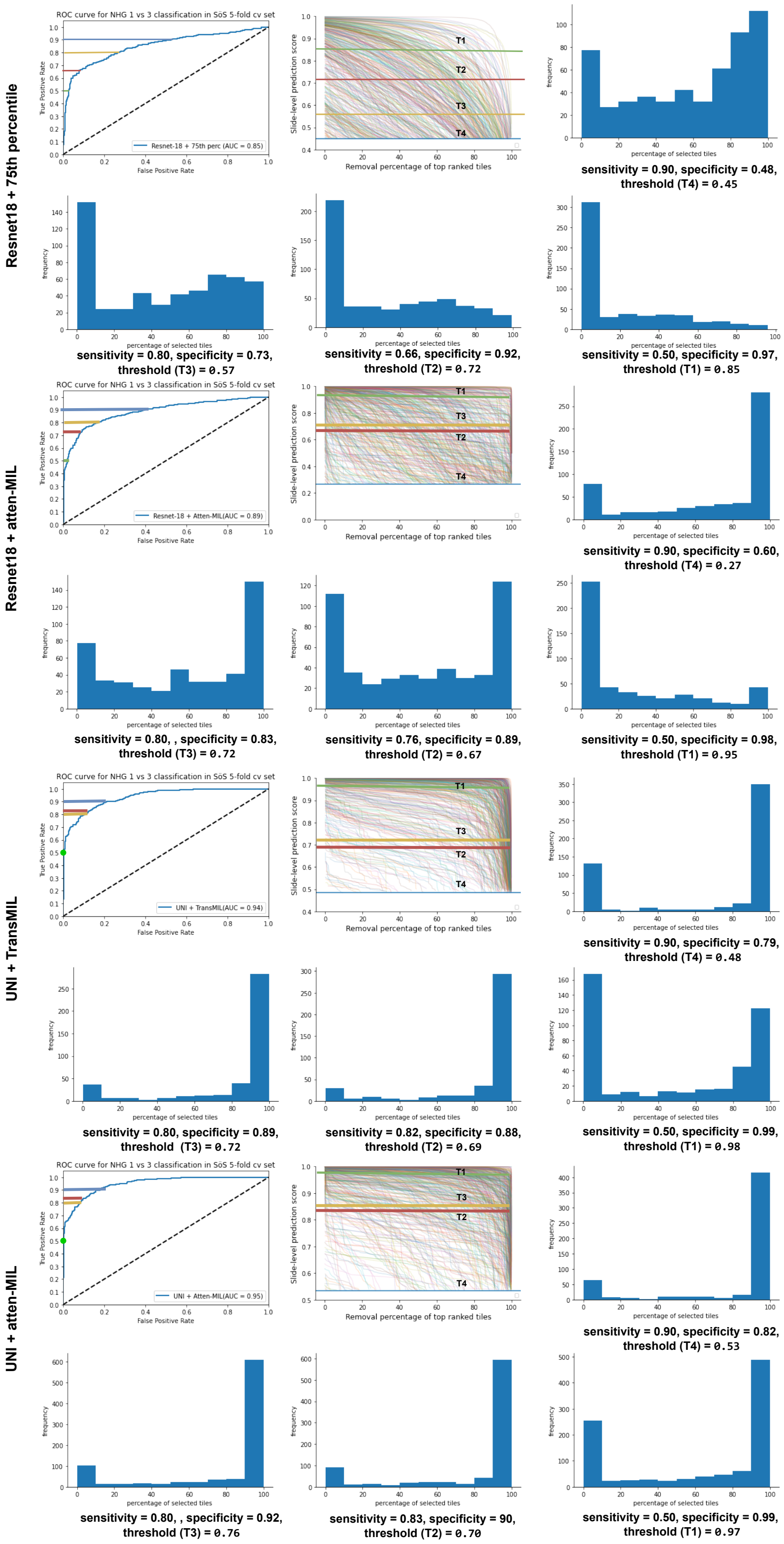

Figure 3: WEEP plots and histograms for different modelling strategies at different classification thresholds corresponding to different model sensitivities: 0.50, 0.80, 0.90, and sensitivity at youden's index, obtained from the ROC curve respectively. Further, histograms show the change in distribution of the percentage of selected tiles for different sensitivity and

classification thresholds. We included the WEEP plots and histograms at classification threshold determined using youden's index in the main manuscript for the all the modelling strategies.

Visualisation of WEEP selected tiles using attention scores from TransMIL and atten-MIL tile-to-slide aggregator model

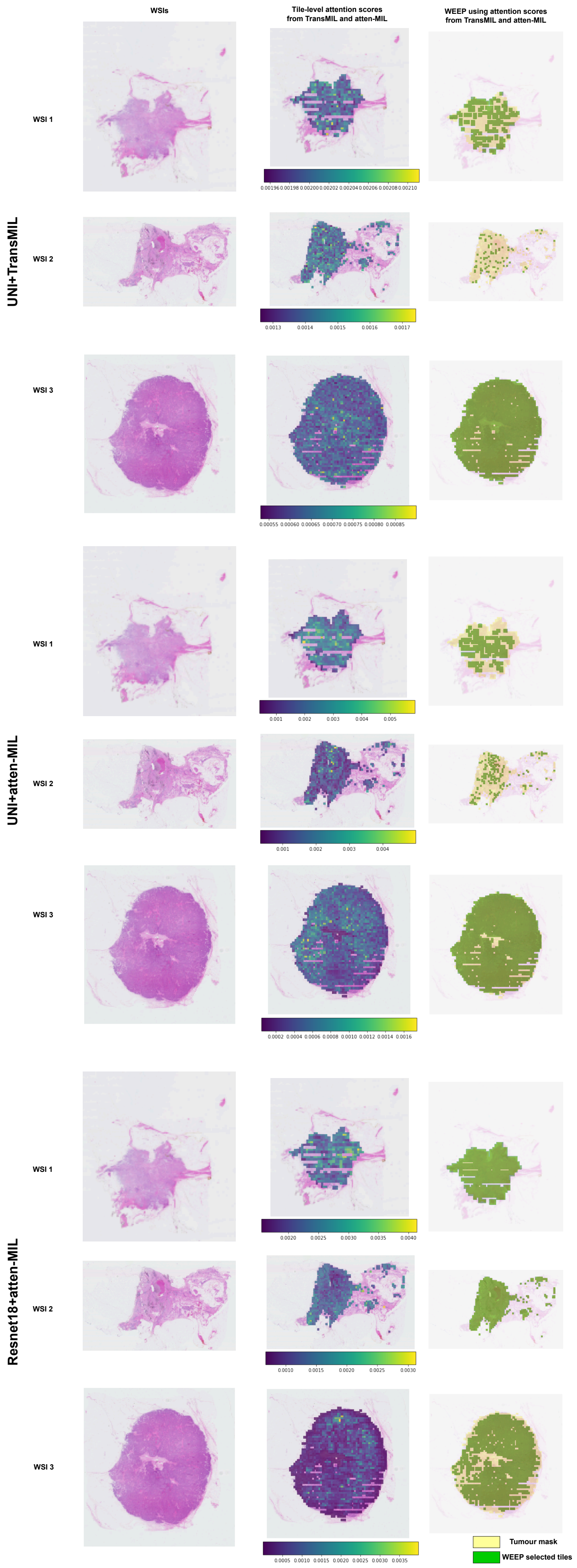
WSIs
Tile-level attention scores
from TransMIL and atten-MIL
WEEP using attention scores
from TransMIL and atten-MIL
UNI+TransMIL
WSI 1
0.00196 0.00198 0.00200 0.00202 0.00204 0.00206 0.00208 0.00210
WSI 2
0.0013 0.0014 0.0015 0.0016 0.0017
WSI 3
0.00055 0.00060 0.00065 0.00070 0.00075 0.00080 0.00085
UNI+atten-MIL
WSI 1
0.001 0.002 0.003 0.004 0.005
WSI 2
0.001 0.002 0.003 0.004
WSI 3
0.0002 0.0004 0.0006 0.0008 0.0010 0.0012 0.0014 0.0016
Resnet18+atten-MIL
WSI 1
0.0020 0.0025 0.0030 0.0035 0.0040
WSI 2
0.0010 0.0015 0.0020 0.0025 0.0030
WSI 3
0.0005 0.0010 0.0015 0.0020 0.0025 0.0030 0.0035
Tumour mask
WEEP selected tiles

Figure 4: Visualisation of WEEP selected tiles using attention scores from TransMIL and atten-MIL tile-to-slide aggregation models with UNI and fine-tuned Resnet-18 as the tile-level feature extractors. The column 1 represents the original WSIs, column 2 includes the heatmap of attention scores for all tiles in different modelling strategies, and column 3 includes the binary mask of WEEP selected tiles overlayed on the tumour mask and the original WSI.

Application of WEEP for histological grade 1 WSIs

We applied the WEEP on histological grade 1 WSIs and observed the WEEP plots and distribution of the percentage of selected tiles (Figure 4). We performed the analysis on the tile-level prediction scores for histological grade 1 from fine-tuned Resnet-18 with 75th percentile as the tile-to-slide level prediction scores.

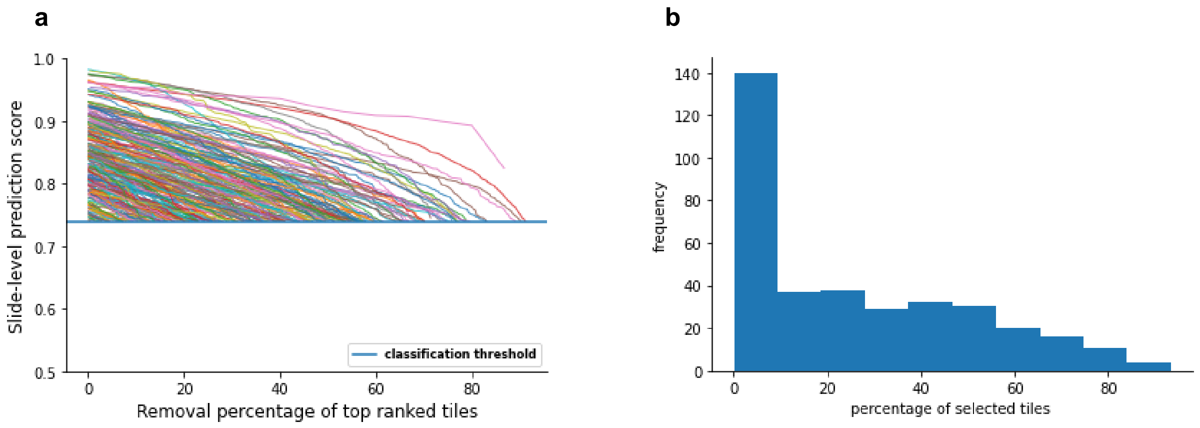

Figure 5: Application of WEEP on histological grade 1 WSIs. **a** WEEP plot demonstrating the backward selection of tiles with decrease in slide-level score. **b** Distribution of the percentage of selected tiles.

References

1. Chen RJ, Ding T, Lu MY, Williamson DFK, Jaume G, Song AH, et al. Towards a general-purpose foundation model for computational pathology. Nat Med. 2024 Mar;30(3):850–62.

2. Sharma A, Weitz P, Wang Y, Liu B, Vallon-Christersson J, Hartman J, et al. Development and prognostic validation of a three-level NHG-like deep learning-based model for histological grading of breast cancer. Breast Cancer Res. 2024 Jan 29;26(1):17.

3. Bottou L. Stochastic Gradient Learning in Neural Networks. 1991 [cited 2022 Nov 14]; Available from: https://www.semanticscholar.org/paper/82eec4af1475de9a7e876bcbaddb4a0c4a1dc187

4. Vallon-Christersson J, Häkkinen J, Hegardt C, Saal LH, Larsson C, Ehinger A, et al. Cross comparison and prognostic assessment of breast cancer multigene signatures in a large population-based contemporary clinical series. Sci Rep. 2019 Aug 21;9(1):12184.
